# Editorial: Organohalide Respiration: New Findings in Metabolic Mechanisms and Bioremediation Applications

**DOI:** 10.3389/fmicb.2019.00526

**Published:** 2019-03-21

**Authors:** Shanquan Wang, Jianzhong He, Chaofeng Shen, Michael J. Manefield

**Affiliations:** ^1^School of Environmental Science and Engineering, Sun Yat-sen University, Guangzhou, China; ^2^Department of Civil and Environmental Engineering, National University of Singapore, Singapore, Singapore; ^3^Department of Environmental Engineering, College of Environmental and Resource Sciences, Zhejiang University, Hangzhou, China; ^4^School of Civil and Environmental Engineering, University of New South Wales, Sydney, NSW, Australia

**Keywords:** Organohalide respiration, *Dehalococcoides*, electron transport chain, reductive dehalogenase, community composition and function, bioremediation

Organohalide-respiring bacteria can utilize several sets of functional enzymes to couple halogen removal with electron transfer from H_2_ or organic matter (e.g., formate and lactate) to organohalides for cell growth (Fincker and Spormann, 2017; Atashgahi et al., 2018). Consequently, microbial reductive dehalogenation basing on the organohalide respiration represents a promising solution for cleanup of persistent organohalide pollutants and plays a pivotal role in the natural halogen cycle (Wang et al., 2018). The key enzymes catalyzing the above-described process are reductive dehalogenases (RDases). Although microbial reductive dehalogenation has been discovered many years ago, advances in characterizing major organohalide-respiring bacteria and in analyzing crystal structures of reductive dehalogenases were made recently (Löffler et al., 2013; Bommer et al., 2014; Payne et al., 2015). These progresses provides unprecedented insights into microbial reductive dehalogenation and brings us to a new stage to realize that there are many puzzles to solve in the mechanistic understanding and in the application of organohalide respiration in bioremediation. Therefore, this research topic was formulated to provide a platform for the publication of updated information and high-quality research papers on *organhalide-respiring bacteria, RDases and associated electron transport chains, dehalogenating microbial community composition and function*, and *organohalide bioremediation*. We received a total of 19 manuscripts, of which 15 were accepted for publication after a rigorous peer review process.

## Organohalide-respiring Bacteria

In an anaerobic microbial consortium, Peng et al. reported that a novel organohalide-respiring bacterium could be involved in debromination of hexabromocyclododecane (HBCD), and no known dehalogenating bacteria were detected in the consortium with PCR-based analysis. Moreover, with *Dehalococcoides mccartyi* type strain−195, Zhong et al. found the diastereoisomer-specific biotransformation of HBCD with a debromination rate order of α-HBCD > β-HBCD > γ-HBCD. In addition to HBCD, Zhao et al. comprehensively reviewed bacterial lineages and pathways for the metabolic debromination of polybrominated diphenyl ethers (PBDEs). These studies provided interesting information on the organohalide-respiring bacteria of new lineages and novel dehalogenation specificities of known players in microbial reductive dehalogenation.

## RDases and Associated Electron Transport Chains

Many reductive dehalogenase homologous (*rdh*) genes have been identified from a variety of dehalogenating bacteria. Nonetheless, most of *rdh* gene-encoding RDases have not been functionally characterized, which is mainly due to the difficulty in heterogeneous expression of the *rdh* genes. Nakamura et al. reported the successful expression, purification and characterization of PceA encoded by *pceA* from a *Geobacter* strain. Notably, PceA was purified and denatured under aerobic condition, which could be refolded in the presence of FeCl_3_, Na_2_S, and cobalamin under anaerobic condition to recover its PCE dechlorination activity. In view of organohalide respiration, the respiratory electron transfer chain in *D. mccartyi* was elucidated for the first time in Lorenz Adrian's lab, which is surprisingly a protein-dependent chain obviating quinone involvement (Kublik et al., 2016). From the same lab, Seidel et al. further provided insightful information on the participating protein subunits and their interactions of the multi-protein RDase complex mediating the electron transfer from H_2_ to organohalides in *D. mccartyi* CBDB1. By contrast, in the quinone-dependent electron transport chains of organohalide-respiring *Dehalobacter*, Buttet et al. employed multiple strategies to show that PceC, originally proposed to be a transcriptional regulator, might be involved in electron transfer from the quinol pool to RDases. These studies provided very important information fulfilling the knowledge gaps in understanding the organohalide respiration process and the major participating enzymes.

## Dehalogenating Microbial Community Composition and Function

*Dehalococcoides* and other obligate organohalide-respiring bacteria generally need to work closely with other microorganisms for efficient dehalogenation in dehalogenating microbial communities. Chau et al. showed that dechlorination rates in co-cultures were enhanced two- to three-fold compared to *Dehalococcoides* pure cultures, in which the syntrophic partners consumed carbon monoxide (CO) generated by *Dehalococcoides* and consequently relieving CO autotoxicity. Moreover, multiple dehalogenating populations may co-exist in dehalogenating communities and at sites undergoing bioremediation, and many factors can drive their population changes. Pérez-de-Mora et al. showed that chloroethenes as electron acceptors was a major driving force behind *D. mccartyi* population selection and succession in KB-1 cultures and remediation sites. Also, Wang et al. showed the significant relationship between microbial community structure and PBDEs in different mangrove, marine and freshwater sediments. In addition to organohalides as electron acceptors, Xu et al. showed that the presence of other alternative electron acceptors (i.e., sulfate and nitrate) had inhibitory effects on the organohalide respiration. Therefore, these experimental evidences may provide very useful information to guide the optimization of dehalogenating microbial communities for successful bioremediation of organohalide pollutants.

## Organohalide Bioremediation

To enhance the efficiency of remediation, a variety of strategies have been tested, including adding organic polymers and electron shuttling materials. Matturro et al. showed very interesting information on the coupling of organohalide-respiring bacteria with bioreactor operation to tackle problems in organohalide bioremediation. For example, amendment of poly-3-hydroxybutyrate (PHB) in a pilot-scale PHB reactor was shown to effectively stimulate the growth of *Dehalococcoides* and consequently enhance the complete dechlorination of chlorinated solvents at contaminated sites. But amendments may not always work as expected. Zhu et al. demonstrated that the addition of biochar as an electron shuttle enhanced iron and sulfate reduction but decreased dechlorination of pentachlorophenol (PCP). Co-existing organohalides generally have inhibitory effects on organohalide respiration. Wen et al. provided experimental evidence showing inhibitory effects of trichloroethane and triclocarbon on trichloroethene dechlorination. Moreover, the presence of alternative electron acceptors may inhibit bioremediation of sites contaminated with organohalides. Xu et al. investigated the influence of a common competing electron acceptor, ferric oxyhydroxide (FeOOH), on reductive dechlorination of polychlorinated biphenyls (PCBs) in two river sediments (i.e., Hudson River and Grasse River sediments), and showed the inhibitory effects of FeOOH on PCB dechlorination, but in different manners. For example, complete and moderate inhibition of PCB dechlorination were observed upon the addition of 40 mmole/kg FeOOH in the Hudson River sediment and the Grasse sediment, respectively. Beside these biostimulation and bioaugmentation strategies, molecular tools may help improve the management of sites undergoing bioremediation. Lu et al. proposed the obligate organohalide-respiring bacterium, *D. mccartyi*, as a potential biomarker for identifying the possible contamination of uncharacterized organohalides in environmental samples, which could complement current chromatography-based tools in testing and analyzing organohalide contamination.

We want to thank all the authors and reviewers for their valuable contributions to this research topic, and we hope that this collection of reviews and original research articles will be helpful for researchers, engineers and students seeking information about microbial reductive dehalogenation and remediation applications.

## Author Contributions

All authors listed have made a substantial, direct and intellectual contribution to the work, and approved it for publication.

### Conflict of Interest Statement

The authors declare that the research was conducted in the absence of any commercial or financial relationships that could be construed as a potential conflict of interest.
